# A framework for constructing insect steering circuits

**DOI:** 10.1371/journal.pcbi.1014009

**Published:** 2026-04-08

**Authors:** Robert Mitchell, Barbara Webb

**Affiliations:** School of Informatics, The University of Edinburgh, Edinburgh, United Kingdom; Salk Institute, UNITED STATES OF AMERICA

## Abstract

Insects perform a variety of goal-directed navigation behaviours, in which steering is controlled by a comparison between their current and desired heading direction. Recent work has uncovered the details of such a steering circuit in the fruit fly *Drosophila melanogaster*. Here we analyse the principles behind the neuroanatomy and physiology of this circuit to derive five general rules which can be used to construct a class of steering circuits which operate in the same way. These rules are surprisingly permissive, suggesting that across insect species, steering circuits may have differing wiring while remaining functionally identical. We simulate several examples, including an irregular circuit that conforms to the rules, and circuits that break the rules, and examine their ability to control steering towards a varying goal direction. We argue that the principled approach we apply here could be applied more generally in performing comparative analyses in neuroscience.

## Author summary

Steering towards a goal is a fundamental animal behaviour. In fruit flies, the brain circuits underlying this behaviour appear to operate by comparing the animal’s current heading with a desired goal direction, where both angles are encoded in activity across a neural array. Here, we consider the geometric principles needed to obtain reliable steering output for any goal direction from such an encoding. This leads to a set of rules governing steering circuit construction. This framework can be used to construct steering circuits which vary significantly in structure, yet perform the task in the same way. We demonstrate this by simulating example circuits which are shown to be functionally equivalent, while being structurally unique. We also explore cases where the rules are broken, which impairs a circuit’s ability to steer. We argue that this principled or ‘rule-based’ approach to analysing neuroanatomy can help to highlight the features of a neural circuit which make it functionally distinct, and is hence valuable when examining the similarities and differences between the neuroanatomy of different species.

## Introduction

Insects perform various goal-directed behaviours including maintaining a straight-line direction of motion [1], path integration and vector navigation [2], and long distance migration [3]. Each of these requires the animal to know the difference between the direction it is currently facing and the direction it wants to go. One way of obtaining this difference is to neurally encode both its current direction and the desired direction in a common spatial framework, then compare the two [4–6].

The insect brain region known as the central complex contains neural populations in which the location of a sinusoidal bump of activity tracks the current head direction of the animal, often described as an internal compass [7]. Here we take the term compass to mean that, over behaviourally relevant time scales and distances, the activity of each neuron can be consistently associated to the same azimuthal direction with respect to the surrounding environment (noting that this consistency need not necessarily persist across behavioural bouts). Recent work in the fruit fly *Drosophila melanogaster* [5,6] has uncovered a further set of neurons in the central complex which appear to encode the animal’s goal direction with respect to its internal compass. As described in more detail in the following, these neurons modulate the relative activity of steering neurons which act in pairs to drive left/right turns that alter the heading direction of the animal. This can be conceptualised as moving the phase of the compass bump to a specific (goal) position [5,6] such that the animal is facing in the direction of its goal.

Here we examine the geometry underpinning sinusoidal population codes, in combination with functional insights provided by a recent anatomical model [5], to derive a set of five rules which can be used to construct a wide family of steering circuits. While varied in structure, these circuits all operate using the same principle through a combination of compass, goal, and steering neurons by following that basic rule-set. Such rules are useful in producing bio-inspired models but also have relevance in comparative neuroanatomy; different insects may have steering circuits which are anatomically distinct but functionally identical. This is demonstrated by comparing selected steering circuits in simulation. In addition, we simulate rule-breaking circuits, illustrating the ways in which breaking the rules can degrade the ability of the circuit to track the goal. We argue more broadly that such a principled approach to circuit analysis is useful in determining which features of a neural circuit are critical to its function.

## Results

### Deriving our rules

#### Encoding of angles.

Our core assumption in the following, consistent with observations in both insects and vertebrates, is the existence of a set of neurons where the activity of each neuron is consistently associated to a specific azimuthal direction (its “preferred firing direction” or PFD). A given input angle (e.g., the heading of the animal) determines the firing rate of each directionally tuned neuron according to some function of the difference between the input angle and the PFD. For example, in various models of insect neural circuits, the activity is determined by a phase-shifted cosine (e.g., [5,8,9]). This cosine calculation is equivalent to projecting a unit vector with the input angle onto a (unit) basis vector with an angle given by the PFD of the neuron. Given the set of neural activities, the angle can be decoded by using them as coefficients for the basis vector set defined by the corresponding PFDs and taking a population vector average (PVA) [5,7,10–12].

By thus considering a set of neurons as equivalent to a set of basis vectors, we can make some general observations relevant to the layout of PFDs required to encode all azimuthal angles. Beginning with basic concepts to make the connection between geometry and neural encodings clear, a basis of 2D vectors with real components can describe any point in space. For example, if


x=au+bv

then we can choose *a* and *b* such that **x** can be any point, so long as **u** and **v** are linearly independent (i.e., not parallel).

**Fig 1:**
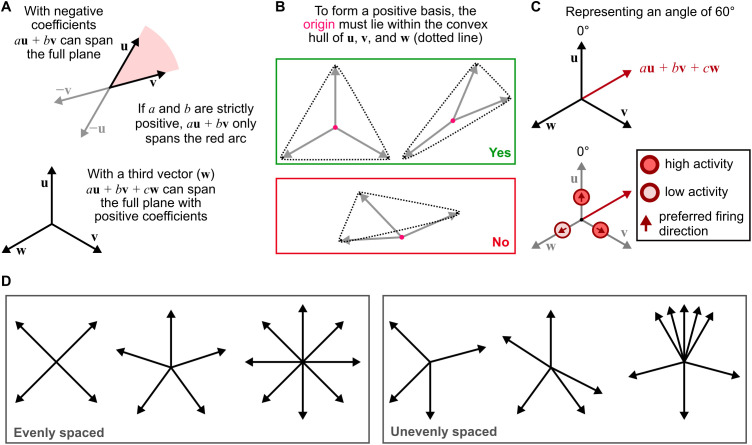
Positive bases. **(A)** Linear combinations of basis vectors can be used to span the plane. However, if negative values are prohibited (e.g., in a neural substrate), a third vector must be included to complete a *positive basis*. **(B)** To determine whether a set of vectors represents a positive basis, we can check whether the origin lies within the convex hull of the vectors. The examples in the green box both represent positive bases. The example in the red box cannot represent any angles which lie outwith the border vectors (using a population vector average). **(C)** Translating the positive basis concept into a neural population. Basis vector coefficients are expressed as relative firing rates for a population of neurons which represent the basis vectors, with angles corresponding to their preferred firing (or associated) direction. So long as these create a positive basis, the population can encode any angle. **(D)** Examples of valid neural bases with both even and uneven spacing. Note that the evenly spaced, four-vector example has no redundancy – removing any of the vectors will break the basis. In the uneven four-vector case, the two lower vectors are redundant, meaning either could be removed without breaking the basis. In the eight-vector example, uneven spacing could be exploited to increase the resolution in one part of the angular domain (for a neural representation).

A *positive basis* is an analogous concept in which the vector coefficients (*a* and *b* above) are restricted to positive values. This means that at least three vectors are required to span the plane (see Fig 1). Now we have,


x=au+bv+cw

with *a*, *b*, *c* all strictly positive. In order to form a positive basis, the origin must lie within the convex hull of **u**, **v**, **w**. Equivalently, the inner angles between each adjacent vector pair must be < 180°, and the sum of the inner angles must equal 360°. The positive basis concept is illustrated in Fig 1.

On the assumption that neurons can only encode positive values in their firing rate (although see [13]), this implies a minimal requirement for the set of PFDs in a neural compass to encode all azimuths is that they form a positive basis. However, there is no requirement that neural tuning curves tile space evenly so long as a positive basis is formed. This means that, for example, a circuit could be robust to uneven spacing arising from errors in construction. Uneven spacing might even be useful: by improving redundancy in small circuits (Fig 1d, compare evenly spaced and unevenly spaced four-vector examples); or increasing resolution in a certain part of the angular domain (Fig 1d, unevenly spaced eight-vector example). Geometrically, the number and distribution of basis vectors used (provided they form a positive basis) makes no difference to the precision of angular encoding, but realistic biological noise and non-linearities that distort the projection could introduce additional constraints on the precision that can be obtained for any particular basis vector set [14].

Note that this is only one possible scheme for neural encoding of angles. An angle is a scalar value so potentially could be encoded in a single neuron’s activity so long as every input angle generated a sufficiently unique firing rate, and downstream neurons were able to decode that firing rate. However, the (positive) basis vector encoding we describe here appears to be that which is in use in the insect brain [7,10–12] and in neuron populations relevant to our discussion [5]. One advantage it might offer is that explicit decoding of the angle is not required for downstream circuits to use the information in useful functions such as steering towards a goal.

### A minimal steering circuit example

**Fig 2:**
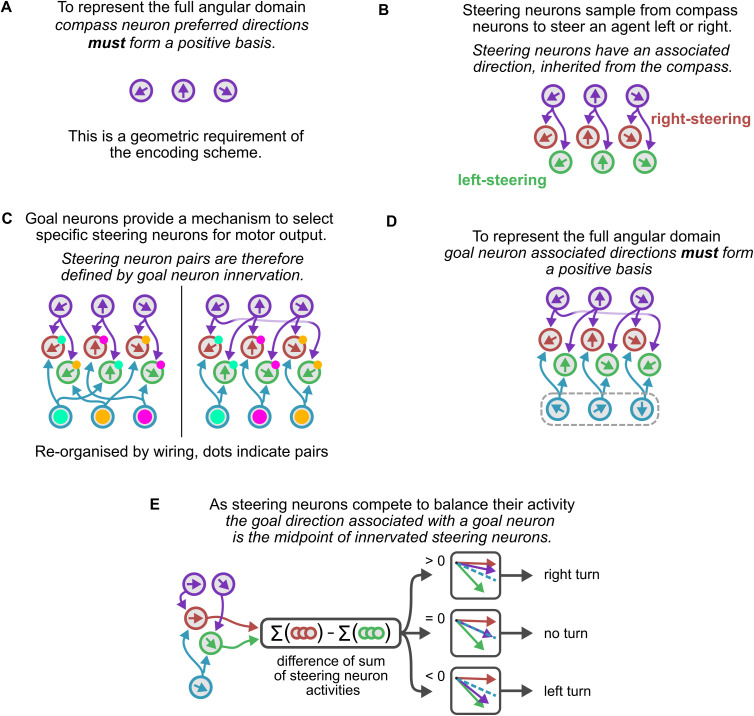
Constructing a minimal example circuit. A schematic of the connectivity, where lines between neurons indicate weighted excitatory connections (see methods for the specific neural and synaptic models used in simulations). Line colour indicates the source (e.g., compass neurons are purple and purple lines come from compass neurons). Arrows within neurons indicate their preferred firing (or associated) direction. **(A)** Compass neuron preferred firing directions must form a positive basis to represent the full angular domain. **(B)** Steering neurons have an associated direction which is inherited from the compass neurons to which they are connected. Steering neurons split into two sub-groups, left-steering (shown in green) and right-steering (shown in red). **(C)** Goal neurons connect to steering neurons and these connections functionally organise the steering neurons into left/right pairs (here indicated by coloured dots). **(D)** As with compass neuron PFDs, goal neuron associated directions should also form a positive basis. **(E)** The steering mechanism functions by taking the difference of the sum of left and right steering neuron activity. If this difference is positive the circuit will generate a right turn, equal will generate no turn, and negative will generate a left turn. This implies that a goal neuron’s associated direction is the midpoint of directions of the innervated steering pair.

To understand the principle of the insect steering circuit, we will start by considering a minimal circuit that encapsulates the core function: to produce a signal indicating which direction to turn to reduce the difference between the current heading direction and a goal. In the following section we will generalise to a range of possible circuits with equivalent function. This construction and its generalisation are based on the geometry described in the previous section, combined with the steering insights provided by [5]. The stages are illustrated in Fig 2.

Compass neurons should be able to represent the full angular domain thus, *compass neuron preferred firing directions must form a positive basis*. The simplest example would be three neurons with evenly spaced receptive fields (Fig 2A).

Following the fly model [5] (also see [8,15]), steering neurons split into left-steering and right-steering populations, which steer the animal by rotating it to balance their activity between two selected points on the compass. In order to do this, steering neurons sample from the compass neurons meaning that *steering neurons inherit an associated direction from the compass*. A simple steering population would have one left and one right steering neuron sampling from each compass neuron (Fig 2B). We then require a way of selecting a left-driving and right-driving steering neuron to work as a pair to drive the agent towards their midpoint (note this implies that the steering neurons in our pair cannot connect to the same compass neuron).

The selection function is provided by goal neurons, each of which should innervate at least one left-steering and one right-steering neuron. Thus *steering neurons are organised into pairs according to goal neuron innervation* (Fig 2C). Further, this implies that *the ‘goal’ direction associated with a goal neuron is the midpoint of the directions of the innervated steering pair* (Fig 2E). That is, activation of a goal neuron will cause left or right steering until the activity of the steering neurons it innervates are balanced, hence their midpoint is the goal direction that this neuron represents.

We now have a set of discrete goal directions which can be considered (as in the compass) as providing a basis for encoding any goal direction in the continuous angular domain, provided *the set of represented goals form a positive basis*. We must choose goal neuron connections such that the set of possible goal directions creates a positive basis. In the simple example illustrated so far, this can be accomplished using three goal neurons, connected to steering pairs as shown in Fig 2D.

Our full minimal example circuit is shown in Fig 2D. It is neat and regular, however the observations we emphasise above generalise to allow circuits which are not neat and regular at all.

### Generalised rules for steering circuits

A steering circuit is made up of three neural populations: compass, goal, and steering; the steering population further splits into *L* and *R* sub-groups. For the interested reader, the analogous anatomical populations in the insect central complex would be EPGs, FC2s, and PFL3s for compass, goal, and steering neurons respectively [5], however we keep our discussion general. Each neuron in the circuit has an associated direction. For the compass neuron PFDs we can in principle choose any set of directions. As illustrated in the minimal example above, the directions associated to the steering and goal neurons are then defined by the circuit structure. Here we describe how, for more complex circuits, these directions are determined, and the constraints this imposes on the connectivity, assuming the desired circuit functionality is to be able to steer from any heading direction towards any goal direction.

We can think of the directions of our neural populations as unit vector sets **C** for compass neurons, **G** for goal neurons, and ${\text{S}}_{L},{\text{S}}_{R}$ for the two sets of steering neurons. We use lower case sub-script to refer to the direction of a specific neuron, e.g., ${\text{c}}_{j}$ is the (vector describing the) PFD of the *j*th compass neuron.

The generalised rules are as follows:

1. **C must form a positive basis.** This allows any heading direction to be represented in the relative activity across the compass neuron population.
2. **Steering neuron direction is a weighted sum of the compass neurons sampled.** In our simple example circuit, each steering neuron received input from one compass neuron, but in principle each could sample from multiple compass neurons in a weighted fashion. The direction associated to that steering neuron is then the weighted sum of the PFDs of all compass neurons sampled: ${\text{s}}_{L,j}=w_{1}{\text{c}}_{1}+w_{2}{\text{c}}_{2}+...+w_{n}{\text{c}}_{n}$ (assume ${\text{s}}_{L,j}$ is then normalised, which also implies that $||{\text{s}}_{L,j}||\neq 0$). While steering neurons also receive input from goal neurons, the direction associated with a steering neuron is defined only by the compass inputs (as the compass sets the spatial reference frame for the rest of the circuit). The sampled compass neurons do not need to be contiguous and different steering neurons could sample from different numbers of compass neurons. The sets of compass neurons sampled by different steering neurons may also overlap.
3. **Steering neurons form left/right pairs according to goal neuron innervation.** Steering pair *j* is defined by the goal neuron ${\text{g}}_{j}$ which provides input to those steering neurons. I.e. ${\text{s}}_{L,j}$ and ${\text{s}}_{R,j}$ both receive input from ${\text{g}}_{j}$. Note that steering pairs do not have to be exclusive. For example, one steering neuron could participate in multiple pairs so long as rules 4 and 5 are obeyed. This means that there could be different numbers of left and right steering neurons. It also implies that goal neurons may innervate multiple steering neurons within the same *L* or *R* sub-population, forming two steering sets rather than strict pairs. The combined direction of one set would effectively be the weighted sum of the directions of all neurons within that set (as in rule 2).
4. **The ‘goal’ direction represented by a goal neuron is defined by the directions of its innervated steering neurons.** The steering mechanism works by selecting neurons from the compass layer and rotating the agent to balance their activity [5]. Goal neuron input to steering neurons moderates their activity and thus acts as the selection mechanism. The goal direction represented by neuron ${\text{g}}_{j}$ can therefore be inferred by examining the connections from compass to steering neurons, and goal to steering neurons. In broad terms, for goal neuron ${\text{g}}_{j}$, its associated direction falls between the directions of the left and right pair of neurons (or sets of neurons), ${\text{s}}_{L,j}$ and ${\text{s}}_{R,j}$, that it innervates. However, there are several factors that affect exactly where it falls; we provide a fully general inference procedure to determine the direction in the Materials and Methods.
5. **G must form a positive basis.** This allows any desired goal direction to be represented by a linear combination of goal neuron activity.

**Fig 3:**
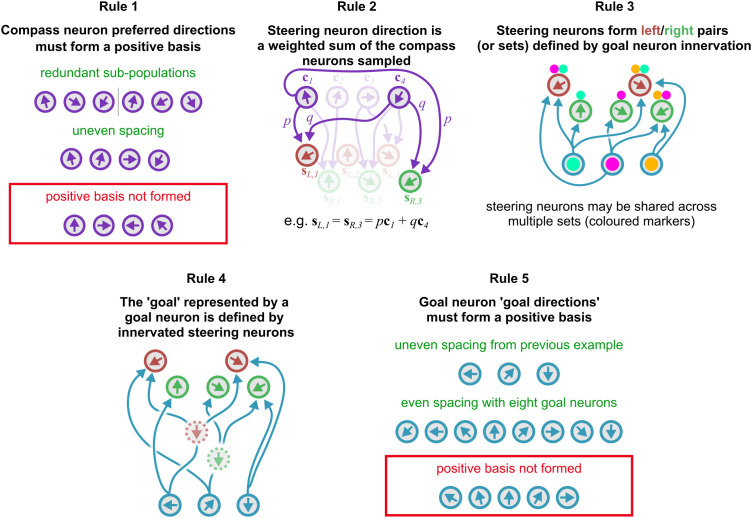
Illustrating our rules for steering circuits. These examples are chosen to illustrate some of the wide range of wiring variation the rules permit. (Rule 1) Compass neuron preferred directions must form a positive basis. The two upper cases form valid positive bases in different ways but the lower case (outlined in red) does not. (Rule 2) Steering neuron direction is a weighted sum of the compass neurons sampled. In our example, we could choose weights *p* and *q* to set the direction to any angle between the sampled compass neurons. (Rule 3) Steering neuron pairs or sets are defined by goal neuron innervation. Coloured markers indicate neurons which are grouped by goal neuron inputs. (Rule 4) The goal direction represented by a specific goal neuron is defined by the steering neurons it innervates. Where a goal neuron inputs to a set of steering neurons within the same steering group (left or right), the direction is defined by a weighted combination of the set (here indicated by a ‘virtual’ neuron with a dashed border). This weighting could also be used to balance multiple neurons in one steering group against a single neuron in the other. (Rule 5) Resulting goal neuron goal directions must form a positive basis. As in the panel for rule 1, two valid cases are given and one rule-breaking case. Note that goal neuron direction is defined by wiring (rule 4), so satisfying this condition requires choosing appropriate connections. Both rules 1 and 5 are geometric requirements of the angular encoding scheme described in existing literature.

Examples for each rule are given in Fig 3 and these examples are deliberately chosen to illustrate the flexibility of the framework. We next consider whether these strange architectural examples can be combined into a functional circuit.

### Demonstrating the functional equivalence of rule-following circuits

We created a simulation in which a model steering circuit is fed a series of goal angles, resulting in a random walk. We simulated the fly circuit model from [5] along with several uniform circuits (with *n* = 3, 5, 8, and 21 neurons, see Materials and Methods). To validate our framework, we also designed an ‘unintuitive’ example circuit (based on Fig 3) for which it should be possible to find working weights.

**Fig 4:**
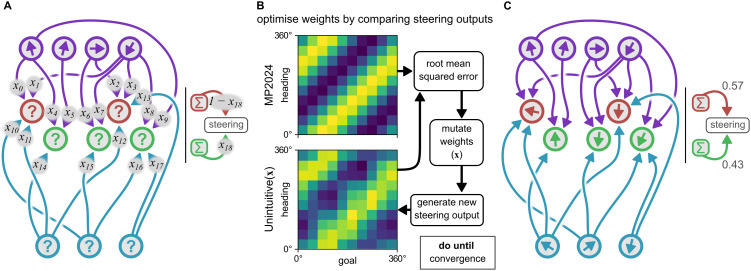
Creating an unintuitive example circuit by comparing the steering output of a given parameterisation to the output from [5]. **(A)** The initial circuit is configured such that there are four compass neurons with preferred firing directions of 350°, 10°, 90°, and 200°. There are two *L* steering neurons and three *R* steering neurons, and three goal direction neurons. A set of connections are chosen which may exist according to a parameter vector **x**. We also include a weight on the steering output meaning that (for example) *L* steering neurons may generate more steering than *R* neurons for the same level of activity. **(B)** The parameter selection process. We first generate the set of steering outputs from the fly model (MP2024) [5] by computing the steering signal for different heading/goal combinations (linearly sampled from 0° to 360°). A parameter vector is generated for the unintuitive circuit, then its steering output computed and compared to the model from [5]. The parameter vector is then mutated and the cycle continues until a minimum root mean squared error is achieved. The mutation process is driven by differential evolution, provided by [16]. **(C)** The final circuit generated by the optimsation process. Despite the strange steering neuron tunings, the goal direction neurons form a positive basis and the circuit can steer (Fig 5).

We call this circuit unintuitive because is layout does not have the regularity we might expect of a central complex circuit, and it is not intuitively obvious that it should work (in contrast to the circuit in figure 2, for example). We defined this circuit with four compass neurons, three left-steering neurons, two right-steering neurons, and three goal neurons. The architecture was chosen so as to highlight the flexibility of the rules, while remaining human-readable. Compass neuron tunings are irregularly spaced (350°, 10°, 90°, and 200°), steering neurons sample from compass neurons irregularly, the *L* and *R* steering populations are of different sizes, and goal neurons innervate steering neurons in an irregular fashion (leading to steering sets of different sizes). Stochastic optimisation (differential evolution) was used to find the weights for all connections such that the steering output of the circuit matched (as closely as possible) that of the [5] fly circuit (Fig 4).

**Fig 5:**
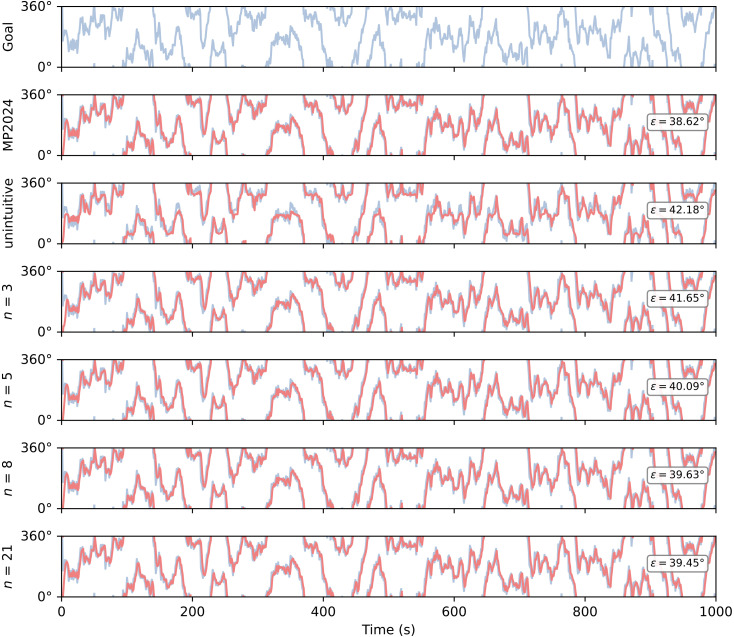
Random walk simulations for rule-following circuits. A random walk is generated by accumulating random samples from a von Mises distribution with μ=0, $\kappa =\frac{1}{0.3}$. This random walk is then fed into the goal neurons for each circuit, and the circuit then steers to follow the walk. The blue line is the random walk over time, the red line is the direction steered by the agent over time. If the circuit is working properly, the lines should overlap. ϵ is the root mean squared (angular) error (RMSE) of the heading against the goal. Lower ϵ indicates that the traces overlap. ‘Goal’ is the angular trajectory the circuits are trying to follow, ‘MP2024’ is the circuit from [5], ‘unintuitive’ is the circuit from Fig 4c, and the remainder are circuits with uniformly spaced compass/goal neuron directions with different numbers of neurons (*n* = 3 is the minimal circuit from Fig 2e). All circuits are roughly tuned to steer at the same rate (between 50° and 60° per second). The κ parameter for the von Mises process was chosen such that the random walk would not change faster than the circuits could possibly follow. All circuits successfully follow the random walk.

The resulting circuit is clearly capable of performing the same function as the uniform circuits, and that of the fly circuit [5] (Fig 5). Though we provide an error metric, performance comparison is not our primary goal at this stage. Here we wish only to demonstrate that the circuits are all capable of completing the task. In the future, it would be interesting to see if evolving circuits with different tasks led to any differences in circuit construction.

### Breaking the rules

To further examine our framework, we set out to design a collection of circuits which broke specific rules in isolation, that is breaking one while following the others. This revealed two classes of rule which we will term prescriptive and descriptive.

Prescriptive rules are those which tell us how we should configure part of the circuit. Rules 1, 4, and 5 are prescriptive. We are able to choose the compass PFDs, whether or not goal neuron directions lie in the correct place (as defined by wiring), and whether goal neuron directions form a positive basis (either by breaking rule 4 or by choosing our connections carefully).

Descriptive rules are those which tell us the effects of our wiring choices. Rules 2 and 3 may be considered descriptive. Rule 2 describes how steering neurons inherit a direction, but this is only ever defined in order to determine the goal neuron direction, it plays no role once the circuit is constructed. The label assigned has no influence on the neuron’s activity (which is not the case for the goal and compass neurons). Similarly, rule 3 describes how goal neuron connections divide the steering neurons into functional pairs. This is simply a generalisation of the mechanism described in the fly [5], changing this would change the steering mechanism such that it was no longer using the same operational principle.

**Fig 6:**
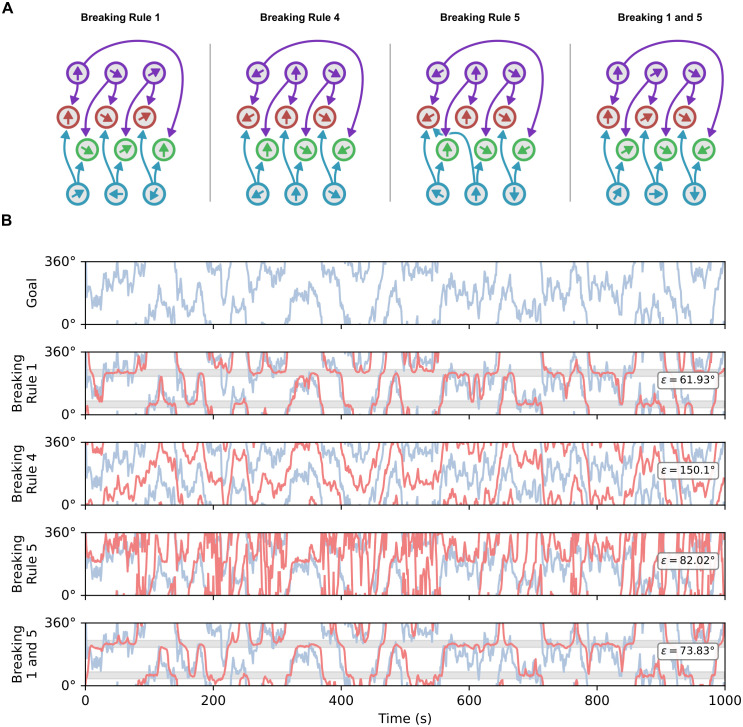
Examining rule-breaking circuits. **(A)** Schematic drawings for rule example rule-breaking models. Rule 1 is broken by choosing PFDs for the compass neurons such that they do not form a positive basis. Rule 4 is broken by arbitrarily setting the associated directions of the goal neurons, ignoring the wiring. Rule 5 is broken by choosing wiring such that the goal neuron associate directions do not form a positive basis. We also provide one additional circuit which breaks rules 1 and 5 together. **(B)** Random walk simulations for rule-breaking models. Rules 2 and 3 are excluded as these are descriptive (see section ‘Breaking the rules’). The error metrics are the same as in Fig 5. ‘Goal’ is the angular trajectory the circuits are trying to follow. The remaining plots correspond with the circuits in panel **A.** The circuit breaking rule 1 partly succeeds in steering towards the goal although there are only certain regions for which it is stable (grey shaded regions). The circuit breaking rule 4 follows the general shape of the goal trace but with a significant offset (the compass and goal neurons are not operating in the same frame of reference). The circuit which breaks rule 5 in isolation fails to follow the goal trace. A final circuit was included which broke both rules 1 and 5 at the same time (neither goal nor compass populations form a positive basis). This circuit appears to follow the goal trace loosely, though it there are significant errors. Again, this circuit appears to have stable headings of approximately 60° and 240° (shaded grey regions).

The key distinction here is that prescriptive rules are those which can be broken. We therefore produced three rule-breaking circuits for rules 1, 4, and 5 (Fig 6A) in isolation, and an additional circuit which broke rules 1 and 5 simultaneously.

### Rule-breaking circuits partly manage the random walk task

Our rule-breaking circuits were given the same random walk task as shown in Fig 5 to see if breaking the (prescriptive) rules led to degradation or disruption of function (Fig 6B).

We broke rule 1 by defining a circuit with three compass neurons, with PFDs of {0°, 60°, 120°}. In theory, the circuit should not be able to represent any compass directions between 120° and 360° (Fig 1). At first glance, this circuit does appear to follow the goal trace, although it struggles to keep up with fast changes (Fig 6B, Breaking Rule 1). On closer inspection, the circuit also appears to switch between two stable regions at approximately 60° and 240°, ignoring shorter turns in the central and border regions of the plot (grey shaded regions, discussed further below).

To break rule 4, we arbitrarily set goal neuron directions to be 180° separated from where they should lie according to the circuit architecture. We would expect this circuit to operate with an offset as the goal and compass neurons are using different frames of reference. As can be seen in Fig 6B (Breaking Rule 4), the circuit executes the correct steering commands but with a significant offset between the goal trace and actual heading trace. It is worth highlighting here that, because the compass and goal neurons are locked into the same reference frame by the steering circuitry, then any changes in the compass reference frame will be reflected in the goal reference frame. Thus, for consistent navigation, the compass reference frame must be stable (see Discussion).

Breaking rule 5 in isolation involved choosing goal neuron connections such that goal neuron associated directions do not form a positive basis. This circuit clearly does not follow the goal trace in general (though there are small portions where the traces appear to match). Based on our geometric starting point, we expected this circuit to fail; however, bearing in mind our seemingly positive result when breaking rule 1, we constructed one additional circuit which broke both rules 1 and 5. This circuit exhibits similar (though noticeably worse) behaviour to that of the circuit which breaks rule 1. Qualitatively, it looks as if the circuit is following the trace, though again it appears to have two stable areas (in approximately the same places at 60° and 240°).

### Rule-breaking circuits do not generate consistent steering output

**Fig 7:**
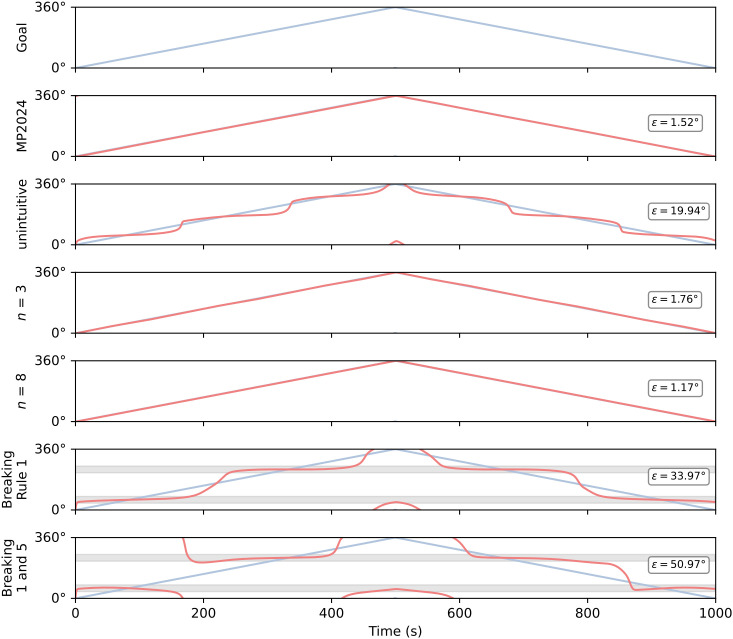
A smooth turn simulation. This test examines how well different circuits cope with different angular regions. (Goal) The goal trace moves from 0 to 360° and back again. The reverse rotation phase was included to see whether turning direction had any effect. (MP2024) The fly circuit from [5] tracks the goal successfully through all angular regions. (Unintuitive) In theory, our unintuitive circuit should also track successfully, but in fact it exhibits a stepped turning regime which approximates the goal but does not match it well. (*n* = 3) This is the minimal uniform circuit presented in Fig 2D. This circuit tracks the goal with comparable behaviour to the fly circuit, as does (*n* = 8), a uniform circuit with higher *n*. (Rule 1) This is the rule breaking circuit shown in Fig 6A (Breaking rule 1). This circuit clearly exhibits two stable headings around 60° and 240°, rather than tracking through all angular regions. These stable headings are indicated by the shaded regions. (1 and 5) This circuit is shown in Fig 6A and breaks both rules 1 and 5. Again, the circuit appears to struggle to track the goal for large angular regions.

The results when breaking rule 1 in isolation, and in combination with 5, were puzzling as there should be large regions of space which the compass and goal neurons cannot represent. The apparent stable headings at 60° and 240° suggested that the random walk may move too quickly over problematic regions of space, effectively masking poor spatial representation as a turn. We therefore conducted a more basic simulation in which the goal signal changed smoothly and slowly in a systematic fashion, allowing us to see if there were particular difficulties with specific regions of space. This simulation is shown in Fig 7.

Both our uniform circuits (*n* = 3 and *n* = 8) and the fly circuit generate consistent turns to track the goal through all angular regions. Interestingly, our unintuitive circuit (which performed well on the random walk task, Fig 5) exhibits a stepped profile though it does approximate the goal through all angular regions. While following the rules has led to a functional circuit, the steering output is not as consistent as that of the uniform or fly models (this can also be seen by looking at the steering output heatmap in Fig 4B). These results suggest that evenly spaced neuron tunings and regular connectivity may be beneficial in a neuron model, even if the underlying geometry implies it should not matter.

The last two traces in Fig 7 show the results for the rule-breaking circuits. They highlight that, while the rule-breaking circuits appeared capable of the random walk task, these circuits do not generate steering commands which are consistent with the required turn. Instead, they exhibit a few stable headings where the circuit effectively does not respond to a change in goal input. The stable headings appear to correspond with the centre of the arc covered by the compass neuron PFDs, and the opposite direction (60° and 240°). Similar results are seen in step change simulations with varying step sizes (S1 Fig, S2 Fig, and S3 Fig).

## Discussion

### Rules for steering circuits

The rules laid out here allow a lot of flexibility in circuit construction. For example:

- The limitations on how steering neurons sample compass neurons imposed by these rules are consistent with a very broad variety of sampling patterns, including uneven and overlapping sampling. The resulting angle between steering neuron directions could be different for different steering pairs, so long as rule 5 is obeyed.
- Rule 5 implies that ‘represented goals’ do not have to be equally spaced, and similarly, rule 1 implies that compass neuron tuning does not have to be equally spaced. Equal spacing may bring benefits (see below), but it is not a geometric requirement of the encoding scheme.
- There is no requirement placed on the number of neurons in each layer. Steering neurons can be shared across different steering pairs so long as rule 5 is obeyed.

While we primarily view such flexibility through a modelling lens, it also permits biological circuits to reconcile angular representations in neuron populations with different numbers of computational units (L. F. Abbott, personal communication). For example, the fly model presented by [5] uses an eight neuron angular encoding in the compass with PFD spacing of 45°, and the goal neurons use a 12 neuron encoding with directional spacing of 30°. The ability to vary sampling strategies in a weighted manner permits the interoperability of these differently sized populations.

As described in Fig 2, a minimal but sufficient circuit could be constructed with three compass neurons, three goal neurons, and six steering neurons (Fig 2D). Interestingly, the minimal circuit is very similar to a model circuit evolved by Haferlach et al. [15]. The only real difference is that their circuit includes speed inputs to both integrate and discharge a homing vector for path integration. In terms of steering principle, they are identical. The same steering mechanism appears in other path integration models [8,17]. A similar fly-inspired steering model has also been constructed that includes an additional set of gain control neurons (PFL2s), which help to alleviate anti-goal fixations [6] (an issue we did not explore here).

The minimal circuit might suggest that the steering circuit anatomy described for the fruit fly [5] is more complicated than it needs to be. However, while all circuits consistent with the stated rules may operate in the same way, they may produce subtle (or not so subtle) variations in behaviour or dynamics. For example, recent work has shown there is a trade-off between the number of computational units in a (ring attractor) compass circuit, and the precision of tuning required for accurate angular representation [14]. Anecdotally, we observed that uniform steering circuits with larger numbers of neurons were more robust to changes in the neural activation function. Thus, while it is possible in principle to have a compass made up of three neurons, this could be more error-prone or more difficult to tune than than one with say, sixteen neurons. It is also possible that a three neuron network (or more generally, circuitry spanning the midline with an odd number of neural computational units) may be less likely to evolve (or harder to produce during development) in bi-lateral animals. Constraints on ancillary connections, such as the ring attractor architecture of the compass in insects, may also place restrictions on the number of compass neurons under certain assumptions [18], or the distribution of PFDs (see below).

Thus, specific details such as the number of each neuron type could be behaviourally relevant, or it could be the result of evolutionary and/or developmental constraints with minimal behavioural impact. Taking a principled (or rule-based) approach to analysing neuromorphology and physiology is helpful in filtering out which properties are likely to be more or less functionally relevant. Two simple examples can be found in the fruit fly circuit described by [5]:

- There are observed wiring patterns such as one steering neuron sampling from multiple compass neurons or different steering neurons sampling from the same compass neurons. This multiple sampling is distinctive and appears to be functionally important, however our rules suggest this is not the case (rules 2, 4, and 5). As we noted above, the wide flexibility of possible sampling patterns allows neural populations of different sizes (encoding the same type of information) to interoperate.
- The compass neurons in their model split into two overlapping neural populations (following the anatomy of the fruit fly). Most preferred directions are represented twice over in the population and some steering neurons sample from different sub-populations. Again, this is a fairly distinct anatomical feature and it is not clear if it is functionally relevant. Rules 2 and 3 tell us how steering neuron pairs are defined, and how steering neuron activity is affected by the sampling pattern. Rules 4 and 5 tell us how we must wire up the goal neurons. Finally, rule 1 tells us that the double representation of preferred directions does not affect the function of the steering circuit.

We should note that the relevance of a given anatomical feature depends on the function under scrutiny. While the double-representation of compass neurons is not important for the steering circuit it could be important elsewhere. For example, in the fruit fly it appears to subserve the mechanism by which self-rotation inputs shift the compass bump [10].

### Rule-breaking circuits respond to input angles outwith their bases

In examining rule-breaking circuits, we found that circuits which did not use positive bases in either the compass, or both compass and goal neuron groups appeared able to perform our random walk task (Fig 6B). On closer inspection however, the random walk task was found to mask a poor angular representation which could be clearly seen in a more systematic simulation (Fig 7). Nevertheless, these circuits were responding to inputs (directions of heading or goal) which, according to our geometric reasoning, they should not have been able to encode.

This unexpected result is in fact a consequence of how we have defined the directional tuning curves for the compass neurons in this work. The combination of using the cosine to define the input (Materials and Methods, equation 2) and the sigmoid firing rate model (equation 1) means that for any given compass neuron, all angles produce some activity level. This broad tuning facilitates continuous encoding of angles with a small number of neurons, but it also produces a unique pattern of firing for every angle even when the compass neuron PFDs do not form a positive basis. The steering mechanism relies on activity imbalances between paired steering neurons, and the tuning curve means that imbalances can be generated through the full angular domain. As a result, the circuits can steer through regions they should not be able to encode (according to our geometric reasoning) although not able to steer well or coherently.

### Evenly spaced basis encodings in insects

Although we have emphasised that any positive basis, including irregularly distributed directions, should suffice for coherent steering, it is in reality the case that angular encodings in the relevant neuron populations do appear to use approximately evenly spaced PFDs which naturally form positive bases [5]. This might provide a functional advantage. A steering circuit should ideally exhibit rotational invariance, that is, generate a consistent steering output wherever the difference between the goal and heading are the same, for any rotation of the animal (L. F. Abbott, personal communication). Based on our results, it seems that evenly spaced neuron tunings lead to better rotational invariance in the steering signal, e.g., we see such invariance in the fly model (Fig 4B, upper heatmap), but less regularity in our unintuitive model (Fig 4B, lower panel, and Fig 7). Thus, a positive basis may be enough to approximate rotational invariance, but evenly spaced neuron tunings enhance this property. Note that breaking rules 1 and 5 significantly disrupts the rotational invariance of the circuit (Fig 7). If even spacing does provide a more consistent angular representation and steering output, then this could provide evolutionary pressure towards circuit architectures which facilitate such a representation.

An alternative explanation is that evenly spaced PFDs are an emergent feature of insect neuroanatomy. In insects the head direction (compass) circuits take the form of a ring attractor [11,19]. These circuits are characterised by local excitation combined with lateral inhibition which creates a stable ‘bump’ of activity in one part of the network. Injecting a small amount of noisy activity into an appropriately tuned ring attractor network can cause a stable bump to form in the absence of any sensory input (see [20, Ch. 7], manuscript in preparation). If we then add a neural population to facilitate angular integration from proprioception (e.g. PENs in insects [10]), then each neuron in the ring will appear to have a preferred firing direction, even though there is no causal external stimulus (e.g., a related visual field [21]). The local excitation and lateral inhibition characteristics of a ring attractor that facilitate smooth movement of the bump would cause these preferred directions to be evenly spaced in a stable circuit.

### Potential for comparative analyses in insects and beyond

Our work is relevant for comparing/modelling steering networks across insect species. We have relied heavily on the example of fruit fly throughout, because it is the only steering circuit that has been functionally characterized in vivo [5]. While the central complex is notable for broad conservation of the circuit architecture across species, minor variations in wiring have been observed and modelled [19]. As we gain model comparative data (see, for example, [22]), the framework presented here can provide an analysis tool to investigate the functional consequences of any differences observed across insect species.

Looking beyond insects, recent work in larval zebrafish has identified a set of putative head direction (HD) cells which appear remarkably similar to the fly head direction system [23]. The zebrafish HD cells appear to implement a ring attractor [23], as is the case in fruit flies [11,19], highlighting the evolutionary significance of this structure for stable angular encodings. Further work has revealed that these HD cells receive visual input in a flexible manner [24], which appears identical in principle to the mechanism described in the fruit fly [7,12,25]. It will therefore be interesting to see if future work reveals a zebrafish steering circuit which is constructed according to the rules we present here.

### Goal neurons and changing reference frames

The analysis presented here has consequences for the way the associated direction for goal neurons is typically treated in models. Similarly to compass neurons, the goal is often defined as a population code across a set of neurons using a cosine activation function:


$$I_{j}=\text{cos}(\theta_{g}-\theta_{j})$$

where *I*_*j*_ is the input to the *j*th goal neuron, $\theta_{g}$ is the goal direction and $\theta_{j}$ is the associated direction of this specific neuron. The obvious next step is to define these associated directions for each neuron in the population, but deceptively, these directions will not be anchored to the external world (as is demonstrated by our rule-breaking circuit for rule 4 - Fig 6).

More specifically, although we might label a goal neuron in our model as having a direction (e.g., of 0°), the actual direction in which the circuit will steer in response to activation of this goal neuron depends on the directions of the steering neurons this goal neuron innervates, which depends on the PFDs of the compass neurons sampled by those steering neurons. Thus, even in a circuit with a perfect geocentric compass we must analyse the wiring to establish what are the anatomically defined directions associated with the goal neurons [5]. Further, in insects, the inputs to the compass are plastic [12,25,26] which (in theory) allows the compass representation to drift or otherwise change with respect to the world. This means the same pattern of goal neuron activity could represent two different geocentric directions, if the compass encoding has changed.

This may not matter for brief goal-directed behaviours such as straight-line orientation [1] (in fact, it could be beneficial if your aim is to be unpredictable [27,28]). However, for an animal navigating to a set location [8,29,30], it is critical for the compass to stay consistent with respect to the world, otherwise the same pattern of goal neuron activation would not point consistently to that location. How the insect stays consistent is, at present, unknown. The compass does have a calibration mechanism [9,31,32], however it is not clear if this mechanism would be sufficient to eliminate all drift (see [20, Ch. 7], manuscript in preparation).

### Conclusion

By combining a recent model of fruit fly goal-directed navigation [5] with elementary geometry underlying angular encodings in the insect brain, we have presented here a set of general rules according to which insect-inspired steering circuits may be constructed. While these rules were derived for modelling purposes, we believe that they may be useful in analysing and comparing real steering circuits. While not without limitations, we believe that this principled (or rule-based) approach to analysing insect neuroanatomy is valuable in distinguishing which morphological characteristics of a circuit make it functionally distinct.

## Materials and methods

### Circuit simulations

#### Rate model.

Neurons are represented using a simple firing rate model.


$$r=\frac{1}{1+e^{-a(I-b)}}$$ (1)

where *r* is the firing rate of the neuron and *I* is the input. The slope and bias parameters are set to *a* = 2 and *b* = 0.6 respectively. The parameters were tuned by hand and the same activation function was used for all neuron types and across different models. A bold **r** is used to denote a vector of firing rates (e.g., **r**_*C*_ is population response for the compass neurons).

#### Compass neurons.

Compass neuron input is


$$I_{C_{j}}=\text{cos}(H-\theta_{j})$$ (2)

where *H* is the agent’s current heading and $\theta_{j}$ is the preferred firing direction (PFD) for the *j*th neuron.

#### Goal direction neurons.

Goal direction neuron input is given by


$$I_{G_{j}}=\text{cos}(G-\phi_{j})$$ (3)

where *G* is the agent’s current goal direction and $\phi_{j}$ is the associated direction of the *j*th goal neuron.

#### Steering neurons and steering commands.

Steering neuron input is


$$I_{S_{L}}=W_{C\to S_{L}}\cdot {𝐫}_{C}+W_{G\to S_{L}}\cdot {𝐫}_{G}$$ (4)

$$I_{S_{R}}=W_{C\to S_{R}}\cdot {𝐫}_{C}+W_{G\to S_{R}}\cdot {𝐫}_{G}$$ (5)

where $W_{P\to Q}$ represents the weight matrix from neural population *P* to population *Q*. The final steering command is given by


$$\text{steering}=k\cdot (\sum {\text{r}}_{S_{R}}-\sum {\text{r}}_{S_{L}})$$ (6)

where *k* is a scaling parameter which determines how quickly the network can steer.

### Uniform circuits

Uniform circuits are made up of *N* compass neurons, *N* goal direction neurons, and 2*N* steering neurons. Compass neuron preferred directions are evenly spaced over 360°


$$\theta_{j}=\frac{360^{\circ }j}{N}$$ (7)

where *j* ranges from 0 to *N* − 1. Steering neurons receive input from compass neurons as


$$W_{C\to S_{L}}=d\cdot {\text{I}}_{N}$$ (8)

$$W_{C\to S_{R}}=d\cdot \text{roll}({\text{I}}_{N},1)$$ (9)

where ${\text{I}}_{N}$ is the identity matrix of size *N*, roll(X,n) is NumPy’s roll operation (which shifts and wraps the columns matrix *X* by *n* positions), and *d* = 0.2 is a scale parameter which determines how much influence the compass neurons have over the steering neurons.

Goal neurons provide input to steering neurons according to


$$W_{G\to S_{L}}=W_{G\to S_{R}}={\text{I}}_{N}$$ (10)

Steering neurons are thus organised into pairs which receive input from neighbouring compass neurons.

### Goal neuron associated direction

The evenly spaced compass neuron tunings and structured columnar input to the steering neurons make it relatively straightforward to infer the goal neuron tuning. The angular tuning for compass neuron *j* can be expressed as a complex number


$${\text{c}}_{j}=e^{i\theta_{j}}$$ (11)

The tunings for each of the steering neurons are then simply


$${\text{s}}_{L}=W_{C\to S_{L}}\cdot \text{c}$$ (12)

$${\text{s}}_{R}=W_{C\to S_{R}}\cdot \text{c}$$ (13)

For these circuits, goal neuron directions lie at the midpoint of the directions associated with the innervated steering pair, so we first work out the corresponding left and right components of the goal neuron direction, then we can sum these to get the final vector pointing along the associated goal neuron direction.


$${\text{g}}_{L}=W_{G\to S_{L}}\cdot {\text{s}}_{L}$$ (14)

$${\text{g}}_{R}=W_{G\to S_{R}}\cdot {\text{s}}_{R}$$ (15)

$$\text{g}={\text{g}}_{L}+{\text{g}}_{R}$$ (16)

Then, remembering that the elements of **g** are complex numbers, $\phi_{j}$ in Eq 3 is given by the complex argument of ${\text{g}}_{j}$.

### Unintuitive circuit

The unintuitive circuit contained four compass neurons, two *L* steering neurons, three *R* steering neurons, and three goal neurons. Compass neuron preferred directions were chosen arbitrarily as {350°, 10°, 90°, 200°} (unevenly spaced but obeying rule 1). Weight matrices were chosen such that some connections definitely did not exist and others could exist with a weight to be determined by an optimisation procedure.

Compass neurons input to steering neurons according to


$$\begin{array}{c} W_{C\to S_{L}}=[\begin{array}{cccc} x_{0} & 0 & 0 & x_{1}\\ 0 & 0 & x_{2} & x_{3} \end{array}] \end{array}$$ (17)

$$\begin{array}{c} W_{C\to S_{R}}=[\begin{array}{cccc} x_{4} & x_{5} & 0 & 0\\ 0 & 0 & x_{6} & x_{7}\\ x_{8} & 0 & 0 & x_{9} \end{array}] \end{array}$$ (18)

Goal neurons input to steering neurons according to


$$\begin{array}{c} W_{G\to S_{L}}=[\begin{array}{ccc} x_{10} & x_{11} & 0\\ x_{12} & 0 & x_{13} \end{array}] \end{array}$$ (19)

$$\begin{array}{c} W_{G\to S_{R}}=[\begin{array}{ccc} x_{14} & 0 & 0\\ 0 & x_{15} & 0\\ 0 & x_{16} & x_{17} \end{array}] \end{array}$$ (20)

Thus, steering neuron associated directions and by extension goal neuron associated directions are unknown until these weights are provided. Finally, we modified the steering output (Eq 6) to include a weight parameter


$$\text{steering}=(1-x_{18})\sum {\text{r}}_{S_{L}}-x_{18}\sum {\text{r}}_{S_{R}}$$ (21)

Functionally, this allows the point at which steering neuron activity is balanced to be tuned such that it does not lie exactly at the midpoint. The inclusion of this parameter in relation to goal neuron direction inference is discussed below.

### Optimisation procedure

In order to find a suitable parameter vector **x**, we linearly sampled angles from 0° to 360° and computed the steering output for every heading/goal combination, using the model from [5]. For a given parameterisation of the unintuitive circuit, we would compute the set of all possible steering outputs in the same way and compute the root mean squared error against the output from [5]. This root mean squared error was minimised using differential evolution [16,33]. Parameters $x_{0}-x_{17}$ were permitted to vary between 0 and 2, and *x*_18_ was permitted to vary between 0 and 1. The bounds for $x_{0}-x_{17}$ were chosen arbitrarily (though positive as these connections appear to be excitatory) and those for *x*_18_ were dictated by Eq 21. The initial state of the network, the optimisation process, and the result are shown in Fig 4.

### Goal direction neuron inference

Inferring the goal neuron direction is complicated by the variability in steering neuron sampling of compass neurons, and goal neuron innervation of steering neurons.

**Fig 8:**
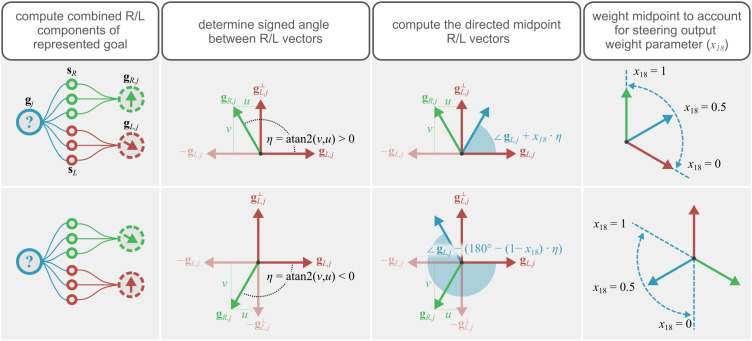
Visualisation of the goal direction neuron tuning inference. The first stage is to compute the combined steering direction for all steering neurons, on the same side, innervated by a goal neuron (Eqs 14 and 15). We then compute the signed angle (η) between the right component and the left component. The directed midpoint is then computed differently depending on whether the signed angle is positive or negative. A positive result indicates that we should use the midpoint of the inner angle, a negative result should use the outer angle, to respect the directionality of the steering neurons. Finally, this midpoint is weighted by model parameter *x*_18_ (Eq 21), to reflect the bias introduced to the goal neurons by the weighted steering output. Rule 1 is guaranteed as we chose the compass neuron tuning, rules 2, 3, and 4 define the goal tuning inference process, and rule 5 is obeyed by the resultant circuit.

Steering neuron directions are computed as in Eq 12 and 13; however in this case, the resultant vectors are all normalised. Similarly, the left and right components of the goal neuron directions are computed using Eqs 14 and 15, with the results being normalised. The normalisation is required as the lengths of the direction vectors can vary, as they are made up of an arbitrary weighted vector sum; subsequent computations assume unit vectors (e.g., Fig 8).

As we cannot make any assumptions about the angle between the left and right components of the goal, the simple vector sum used previously is not sufficient. In the case where the angle between the left and right components is greater than 180°, the represented goal will point in the wrong direction, and if the angle is equal to 180° then the vector sum will give a zero vector. We therefore have to account for the sign of the angle between the left and right components, and work with the angles directly instead of using vectors. A visualisation is given by Fig 8.

For a given goal neuron *j*, combined left-hand component is given by ${\text{g}}_{L,j}$ and the right-hand component by ${\text{g}}_{R,j}$ (Eqs 14 and 15). We begin by computing ${\text{g}}_{L}^{⊥}$, the set of left-hand component vectors which are orthogonal (counter-clockwise) to their counterparts in ${\text{g}}_{L}$. We then compute the projection of ${\text{g}}_{R,j}$ onto ${\text{g}}_{L,j}$ and ${\text{g}}_{L,j}^{⊥}$.


$$u={\text{g}}_{L,j}\cdot {\text{g}}_{R,j}$$ (22)

$$v={\text{g}}_{L,j}^{⊥}\cdot {\text{g}}_{R,j}$$ (23)

$${∠}_{j}=\text{atan2}(v,u)$$ (24)

The final stage gives the signed angle ${∠}_{j}$ between ${\text{g}}_{L}$ and ${\text{g}}_{R}$. In our implementation, *L* steering neurons drive the agent in a positive direction, and *R* steering neurons drive the agent in a negative direction. Thus, the final goal direction can be computed as


$$\eta_{j}=\left\{ \begin{array}{c} \text{arg}({\text{g}}_{L,j})+x_{18}\cdot {∠}_{j},\text{if }{∠}_{j}>0\\ \text{arg}({\text{g}}_{L,j})-(180^{\circ }-(1-x_{18})\cdot {∠}_{j}),\text{otherwise.} \end{array}\right.$$ (25)

and to remain consistent with our complex/vector representation


$${\text{g}}_{j}=e^{i\eta_{j}}$$ (26)

If we limit *x*_18_ to be 0.5, the process amounts to choosing ${\text{g}}_{j}$ or $-{\text{g}}_{j}$, depending on the relative position of ${\text{g}}_{L,j}$ and ${\text{g}}_{R,j}$. If we let *x*_18_ vary, this allows the optimisation procedure to choose where the goal lies between ${\text{g}}_{L,j}$ and ${\text{g}}_{R,j}$ (Fig 8).

As the optimisation procedure is stochastic, it is not possible to concretely state whether or not this extra parameter is necessary. However, it did produce better results with less optimisation attempts. We would therefore tentatively say that, where there are different numbers of steering neurons in each *L*/*R* sub-population, it is likely that this needs to be counterbalanced by weighting motor output. This may be an additional developmental constraint in real insect brains which enforces a degree of symmetry.

### Steering model from [5]

The fly model is implemented using the description given in the paper [5]. Goal (FC2) neuron directions are: −15°, −45°, −75°, −105°, −135°, −165°, 165°, 135°, 105°, 75°, 45°, 15°. *L* steering (PFL3_*L*_) neuron directions are: 67.5°, 22.5°, −22.5°, −22.5°, −67.5°, −112.5°, −112.5°, −157.5°, 157.5°, 157.5°, 112.5°, 67.5°. *R* steering (PFL3_*R*_) neuron directions are: −67.5°, −112.5°, −157.5°, −157.5°, 157.5°, 112.5°, 112.5°, 67.5°, 22.5°, 22.5°, −22.5°, −67.5°. The response for the *j*th steering neuron (in either the *L* or *R* population) is given by


$$S_{L/R,j}=f(\text{cos}(H-\theta_{j})+0.3\cdot \text{cos}(G-\phi_{j}))$$ (27)

where *H* is the current heading $\theta_{j}$ is the direction for the *j*th steering neuron, *G* is the goal direction, and $\phi_{j}$ the associated direction for the *j*th goal neuron. The function *f* is


$$f(x)=29.23\cdot \text{log}(1+e^{2.17\cdot (x-0.7)})$$ (28)

as in [5]. The final steering response is


$$\text{steering}=l\cdot (\sum_{j}S_{L}-\sum_{j}S_{R})$$ (29)

The constant *l* = 0.00018 is used to scale the model output so that it operates on roughly the same timescale as our other models.

### Rule-breaking models

Our rule-breaking models were built by hand and their implementation can be seen by accessing our software repository.

## Supporting information

S1 Fig Step change simulation - 30°. (TIFF)

S2 Fig Step change simulation - 60°. (TIFF)

S3 Fig Step change simulation - 90°. (TIFF)
