# Supplementary figures and images for: A framework for constructing insect steering circuits
Source: PLoS Comput Biol. 2026 Apr 8;22(4):e1014009. doi: 10.1371/journal.pcbi.1014009 (PMC13095111; doi:10.1371/journal.pcbi.1014009)

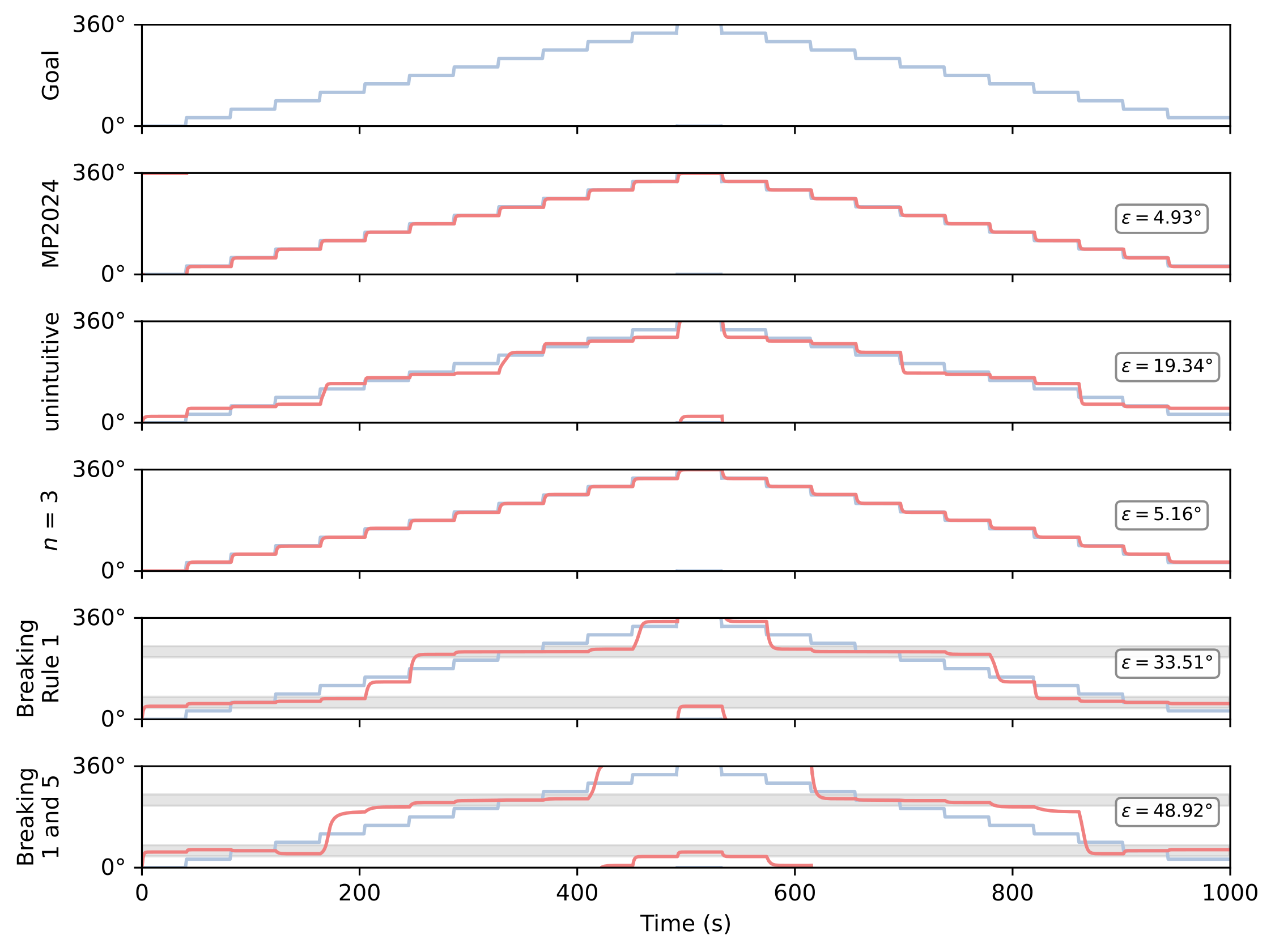

Supplement: S1 Fig — (TIFF) [file pcbi.1014009.s001.tiff]

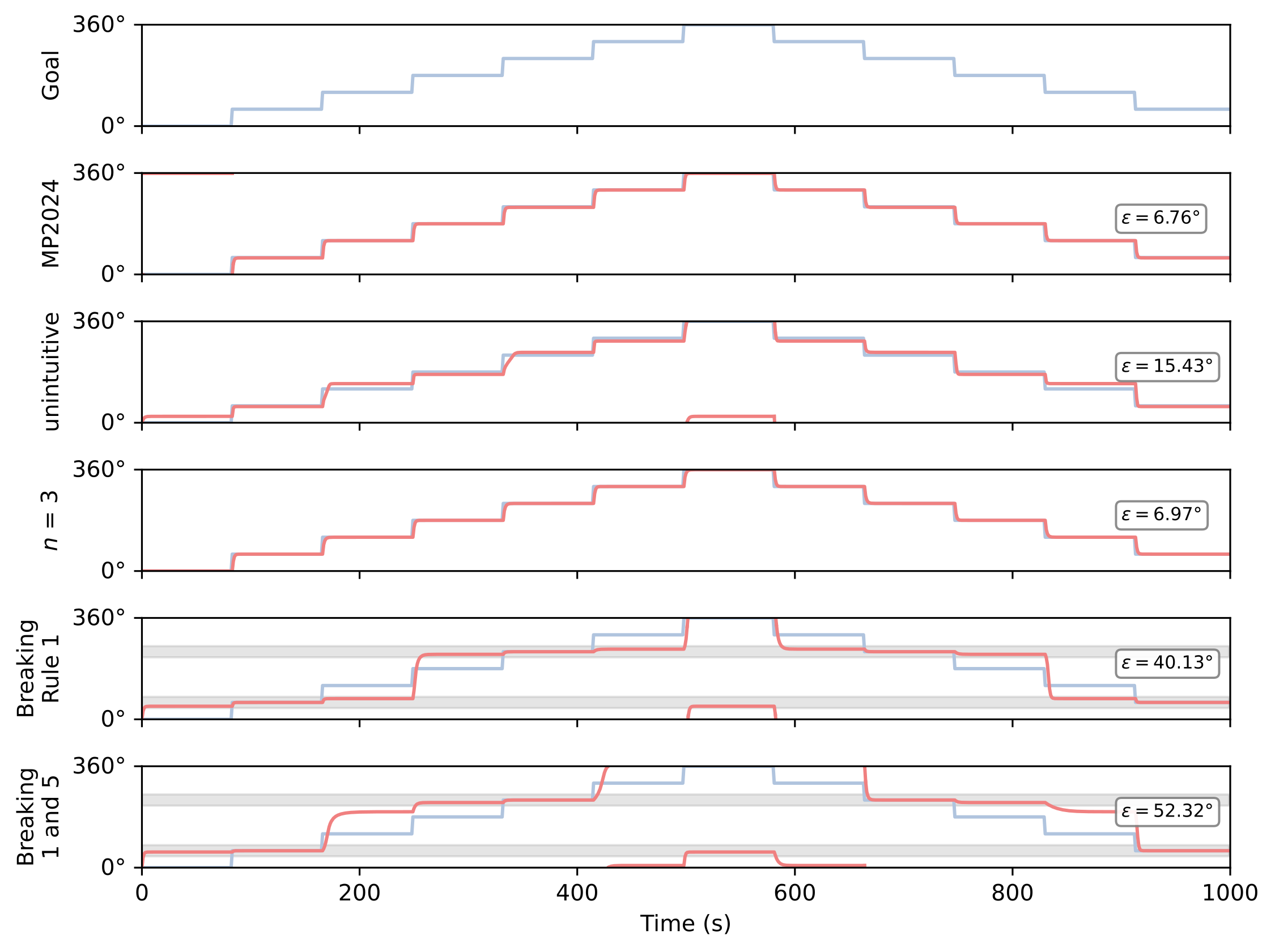

Supplement: S2 Fig — (TIFF) [file pcbi.1014009.s002.tiff]

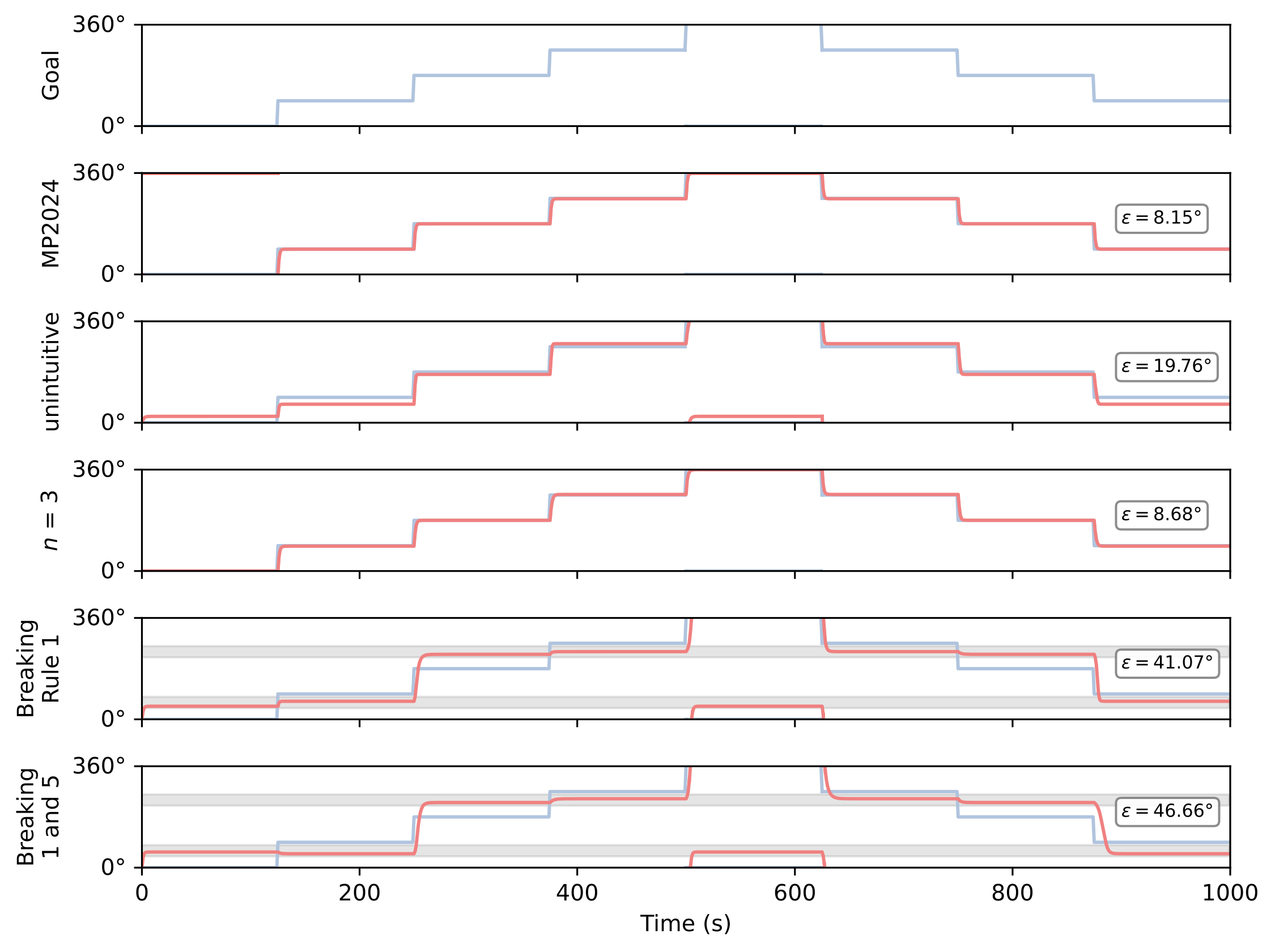

Supplement: S3 Fig — (TIFF) [file pcbi.1014009.s003.tiff]
